# Supplementary material for: Vaccination of SARS-CoV-2-infected individuals expands a broad range of clonally diverse affinity-matured B cell lineages
Source: Nat Commun. 2023 Apr 19;14:2249. doi: 10.1038/s41467-023-37972-1 (PMC10115384; doi:10.1038/s41467-023-37972-1)
Supplement: Supplementary file 1 — Supplementary Information [file 41467_2023_37972_MOESM1_ESM.pdf]

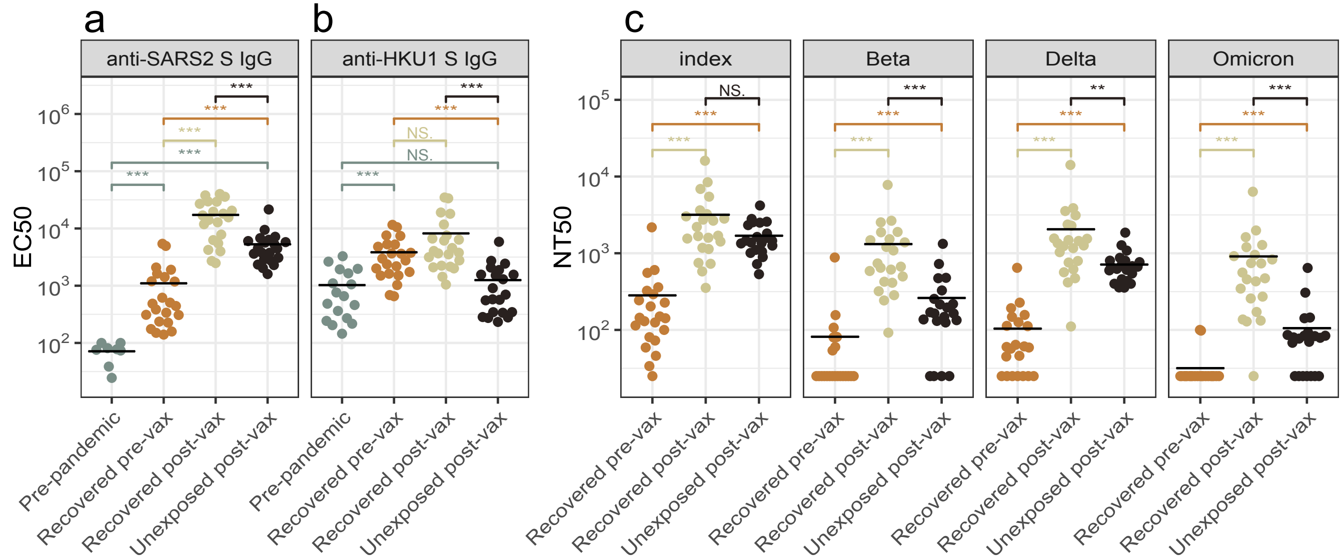

Supplementary Figure 1. Serum IgG titers against SARS-CoV-2/HCoV-HKU1 S glycoproteins and neutralizing antibody titers of donors infected and/or vaccinated against SARS-CoV-2.

(a) Serum IgG titers against the SARS-CoV-2 glycoproteins. (b) Serum IgG titers against HCoV-HKU1 S glycoproteins. (c) Neutralizing antibody titers against index SARS-CoV-2 and variants. Samples were taken from a cohort of individuals a median of 8 days (range: 7-14) days after vaccination by mRNA-1273 (n = 22) or BNT162b2 (n = 21), 22 of whom were previously infected with SARS-CoV-2 (Recovered) and given one dose and 21 who were not previously infected (Unexposed) and given two doses. The previously infected individuals were vaccinated a median of 179 days after disease onset (range: 40-213 days). For these individuals, we also included serum samples collected a median of 42 (range 0-176 days) days before the mRNA vaccination. We used two-sided Mann-Whitney U-tests to compare titers.

*P < 0.05, **P < 0.01, and ***P < 0.001; the exact p-values are provided in the Source Data file.

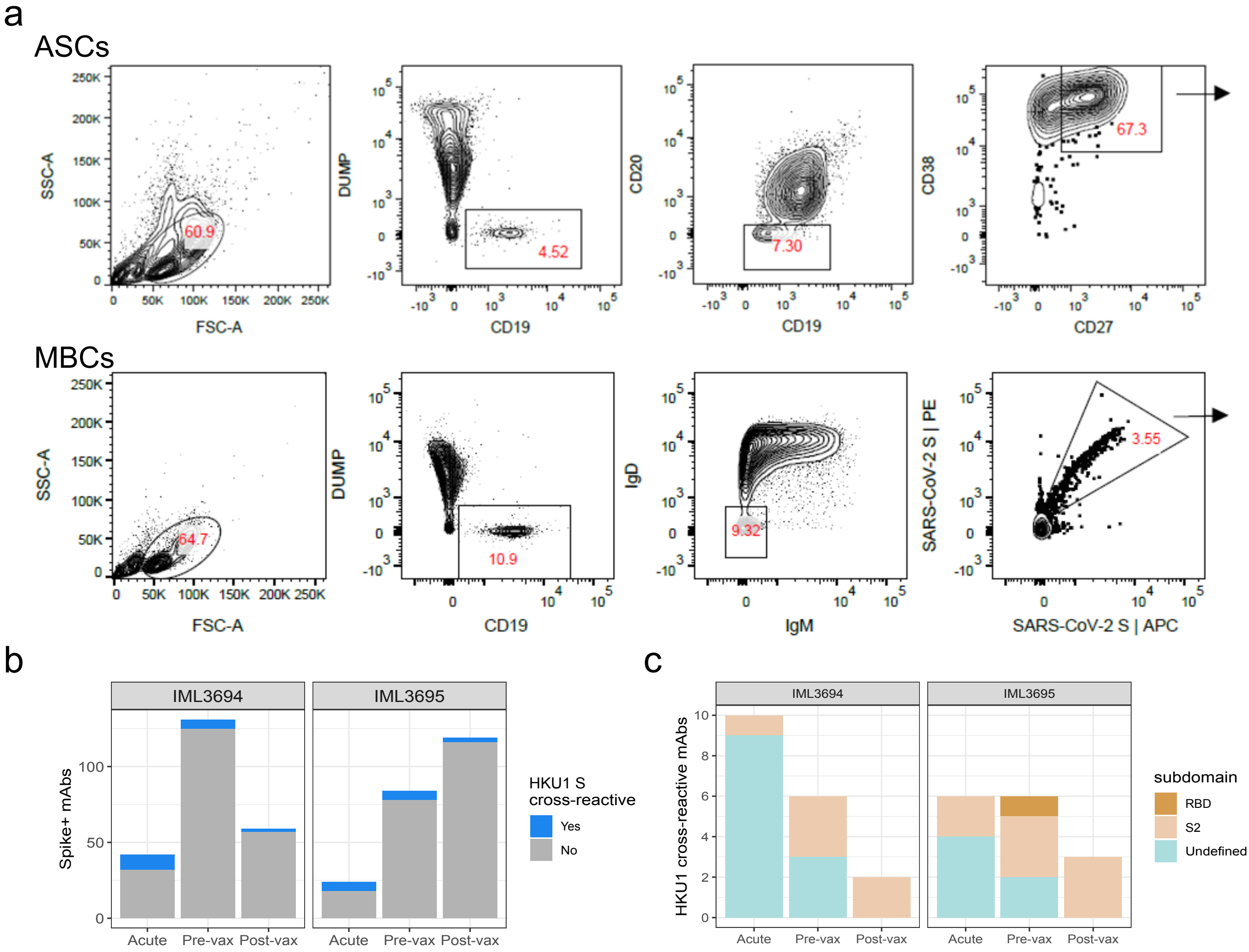

Supplementary Figure 2. FACS strategy and summaries of HKU1 S cross-reactive mAb data.
(a) Flow cytometry plots showing the staining and gating strategy for sorting ASCs and spike-specific MBCs. (b) The number of HCoV-HKU1 and SARS-CoV-2 S cross-reactive mAbs versus SARS-CoV-2 S-specific mAbs isolated at the different time points. (c) Sub-specificities of the HKU1 S cross-reactive mAbs isolated at the different time points.

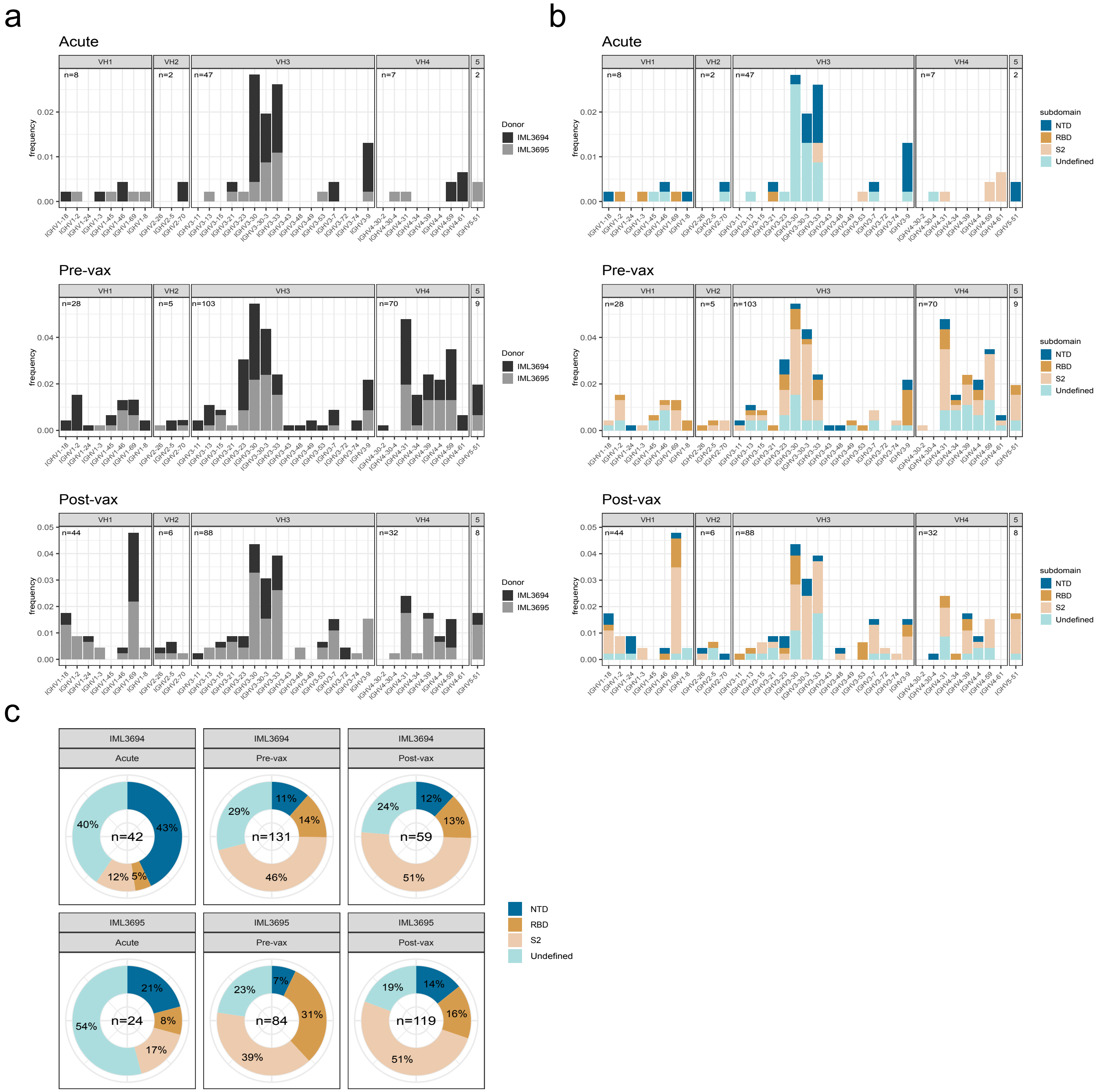

Supplementary Figure 3. Longitudinally separated properties of spike-binding mAbs.
(a) mAb IGHV allele frequencies colored by donor, shown separately for the three sets of mAbs isolated at the different time points. (b) mAb IGHV allele frequencies colored by subdomain specificity, shown separately for the three sets of mAbs isolated at the different time points. (c) Pie charts of mAb subdomain frequencies shown separately for the three sets of mAbs isolated at the different time points.

a

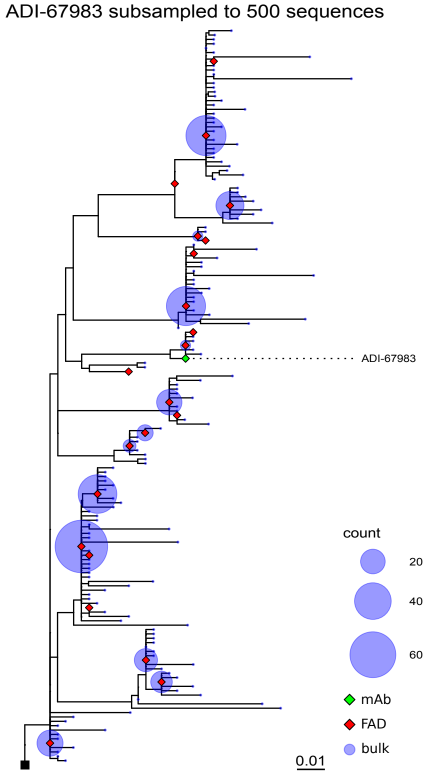

b

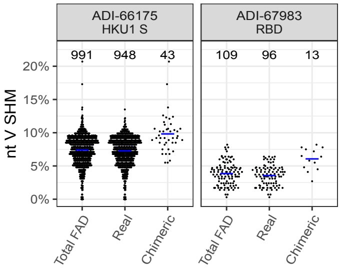

c

Donor	Isotype	Timepoint	Total reads	Merged reads	FAD	Chimera cleaning	Clonotypes
IML3694	IgG	Acute	4842232	4777889	14777	13004	3391
IML3694	IgG	Convalescent	5701290	5586476	13252	12012	4444
IML3694	IgG	Post-vax	8158571	8011800	18038	17204	5627
IML3695	IgG	Acute	4594108	4516270	10072	9016	3027
IML3695	IgG	Convalescent	5620379	5562365	8096	7669	2035
IML3695	IgG	Post-vax	6742232	6632439	17855	17133	3855
IML3694	IgM	Acute	1070302	954167	14501	13948	12126
IML3694	IgM	Acute	652551	606189	23481	23460	21495
IML3694	IgM	Acute	1102412	969176	16930	16678	14979
IML3695	IgM	Acute	921646	829652	13052	12905	10974
IML3695	IgM	Acute	989121	927195	17956	17737	14959
IML3695	IgM	Acute	667778	600625	21124	21102	17881

d

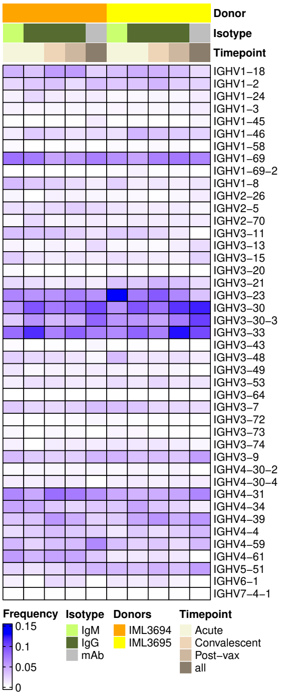

Supplementary Figure 4. FAD and chimera detection sequence pre-processing and sequencing summary statistics.
(a) Maximum likelihood phylogenetic tree of Rep-Seq and mAb sequences belonging to the same lineage as the ADI-67983 mAb prior to FAD denoising, subsampled to 500 sequences. Sequences that remained after Fast Amplicon Denoising are marked in red. The tree is rooted on an inferred germline sequence obtained from the IgBLAST-generated “germline_alignment” column of the sequence with the smallest IGHV SHM in the lineage. (b) IGHV SHM nucleotide percentage dot plots for the ADI-66175 and ADI-67983 clones IgG Rep-seq sequences, demonstrating the difference between SHM distributions with and without chimera removal. Blue crossbars are Rep-seq data averages. (c) Sequence count data at each processing step. (d) Heatmap of clonally collapsed IGHV gene frequencies of bulk libraries and mAbs.
