# Supplementary material for: Vaccination of SARS-CoV-2-infected individuals expands a broad range of clonally diverse affinity-matured B cell lineages
Source: Nat Commun. 2023 Apr 19;14:2249. doi: 10.1038/s41467-023-37972-1 (PMC10115384; doi:10.1038/s41467-023-37972-1)
Supplement: Supplementary file 3 — Description of Additional Supplementary Files [file 41467_2023_37972_MOESM3_ESM.pdf]

File name: Supplementary Data 1
Description: Figure 1 serum titer donor samples datasheet.

File name: Supplementary Data 2
Description: Monoclonal antibody data sheet including sequence data, subdomain specificities, neutralization titers, *VDJ* gene assignment, and primers used to amplify mAbs.

File name: Supplementary Data 3
Description: IgDiscover inferred genotypes for IML3694 and IML3695 as well as primers used for IgM and IgG Rep-seq library preparation.
