# Supplementary material for: Vaccination of SARS-CoV-2-infected individuals expands a broad range of clonally diverse affinity-matured B cell lineages
Source: Nat Commun. 2023 Apr 19;14:2249. doi: 10.1038/s41467-023-37972-1 (PMC10115384; doi:10.1038/s41467-023-37972-1)
Supplement: Supplementary file 7 — Reporting Summary [file 41467_2023_37972_MOESM7_ESM.pdf]

Corresponding author(s): Gunilla B. Karlsson Hedestam

Last updated by author(s): March 14, 2023

Reporting Summary

Nature Portfolio wishes to improve the reproducibility of the work that we publish. This form provides structure for consistency and transparency in reporting. For further information on Nature Portfolio policies, see our Editorial Policies and the Editorial Policy Checklist.

Statistics

For all statistical analyses, confirm that the following items are present in the figure legend, table legend, main text, or Methods section.

n/a	Confirmed	
☐	☒	The exact sample size (*n*) for each experimental group/condition, given as a discrete number and unit of measurement
☐	☒	A statement on whether measurements were taken from distinct samples or whether the same sample was measured repeatedly
☐	☒	The statistical test(s) used AND whether they are one- or two-sided<br>*Only common tests should be described solely by name; describe more complex techniques in the Methods section.*
☒	☐	A description of all covariates tested
☒	☐	A description of any assumptions or corrections, such as tests of normality and adjustment for multiple comparisons
☐	☒	A full description of the statistical parameters including central tendency (e.g. means) or other basic estimates (e.g. regression coefficient) AND variation (e.g. standard deviation) or associated estimates of uncertainty (e.g. confidence intervals)
☐	☒	For null hypothesis testing, the test statistic (e.g. *F*, *t*, *r*) with confidence intervals, effect sizes, degrees of freedom and *P* value noted<br>*Give P values as exact values whenever suitable.*
☒	☐	For Bayesian analysis, information on the choice of priors and Markov chain Monte Carlo settings
☒	☐	For hierarchical and complex designs, identification of the appropriate level for tests and full reporting of outcomes
☒	☐	Estimates of effect sizes (e.g. Cohen's *d*, Pearson's *r*), indicating how they were calculated

Our web collection on statistics for biologists contains articles on many of the points above.

Software and code

Policy information about availability of computer code

Data collection	No software was used.
Data analysis	IgDiscover22 v1.0.0 is available at https://gitlab.com/gkhlab/igdiscover22, scripts used to generate all results in the paper are available at https://gitlab.com/gkhlab/Vaccination_of_SARS-CoV-2_Convalescents and https://doi.org/10.5281/zenodo.7644243. HMM code for chimera identification is available at https://github.com/MurrellGroup/CHMMera/.

For manuscripts utilizing custom algorithms or software that are central to the research but not yet described in published literature, software must be made available to editors and reviewers. We strongly encourage code deposition in a community repository (e.g. GitHub). See the Nature Portfolio guidelines for submitting code & software for further information.

Data

Policy information about availability of data

All manuscripts must include a data availability statement. This statement should provide the following information, where applicable:

- Accession codes, unique identifiers, or web links for publicly available datasets
- A description of any restrictions on data availability
- For clinical datasets or third party data, please ensure that the statement adheres to our policy

Repertoire sequencing data has been deposited to a restricted access repository at Science for Life Laboratory (SciLifeLab) Data Centre with the DOI 10.17044/scilifelab.21518142 to protect donor privacy. Data access requests may be submitted to the SciLifeLab Data Centre at datacentre@scilifelab.se with the DOI to

establish a data sharing agreement. The timeframe for a response to requests is less than a week. The starting database for IGH VDJ genotyping can be downloaded from IMGT V-Quest at https://www.imgt.org/download/V-QUEST/IMGT_V-QUEST_reference_directory/Homo_sapiens/IG/. Source data are provided with this paper.

Human research participants

Policy information about studies involving human research participants and Sex and Gender in Research.

Reporting on sex and gender	The serum IgG titers and neutralizing antibody titer cohorts contained an 8 to 20 and 12 to 22 female to male ratios in the recovered vaccinated and unexposed vaccinated cohorts respectively. The sex of the two lineage tracing donors is not recorded.
Population characteristics	The serum IgG titers and neutralizing antibody titer cohorts had a median age of 63 and 34 for the recovered vaccinated and unexposed vaccinated individuals, respectively.<br>We also have comorbidity data for the infected vaccinated cohort:<br>Comorbidites Proportion of infected donors (%)<br>Diabetes 5<br>Hypertension 45<br>Coronary heart disease 15<br>Chronic lung disorder 25<br>Asthma 25<br>Automimmune disease 20<br>immunsup 10<br>Kidney disease 0<br>Chronic liver disease 0<br>Tumor/cancer 0<br>Dementia 0<br>Other chronic conditions (Allergy, arthrosis, hernia x2, psoriatric athrthritis x2, kidney failure, single kidney after transplantation, hyperlipidemia, pneumocistis pneumonia) 40
Recruitment	The results shown in Supplementary Fig. 1A were performed under permit 2021-00055 and amendment 2021-01387 approved by the Swedish ethics review authority and is part of an ongoing observational clinical trial to investigate immune responses to Covid-19 vaccination (EudraCT number 2021-000683-30). Inclusion of individuals into our prospective open cohort was through informed individual consent. At inclusion, we excluded individuals with medication or comorbidities that have an obvious effect on the immune system (i.e., immunosuppressive medication or pid's). Hence, this minimizes the potential of self-selection bias that can affect results in the study. For the unexposed-vaccinated group, we excluded individuals with self-perceived (and/or diagnosed) SARS-CoV-2 infection. The study participants did not receive any compensation and sex was self-reported. Two other SARS-CoV-2-infected patients were recruited for in-depth longitudinal studies, both who gave informed consent in accordance with the Dartmouth-Hitchcock Hospital (D-HH) Human Research Protection Program (Institutional Review Board) and approved by the Swedish ethics review authority, permit 2021-01850. SARS-CoV-2 infection was confirmed by reverse transcriptase polymerase chain reaction (RT-PCR) after nasal swab in October 2020. Participants received the first dose of the mRNA-1273 vaccine (Moderna) approximately 5 months after the first positive test. Blood samples were collected and fractionated by the Clinical Research Unit of D-HH to obtain PBMCs and serum. The two SARS-CoV-2 infected patients were recruited due their manageability of symptoms, not requiring hospitalization and willingness to provide blood samples at requested timepoints. The age of both donors is on the higher end of typical "adult" age-classification, thus increasing possibility of prior exposures to circulating seasonal endemic coronaviruses.
Ethics oversight	The results shown in Supplementary Fig. 1A were performed under permit 2021-00055 and amendment 2021-01387 approved by the Swedish ethics review authority and is part of an ongoing clinical trial to investigate immune responses to Covid-19 vaccination (EudraCT number2021-000683-30). Two SARS-CoV-2-infected patients were recruited for in-depth studies. Both volunteers gave informed consent in accordance with the Dartmouth-Hitchcock Hospital (D-HH) Human Research Protection Program (Institutional Review Board) and approved by the Swedish ethics review authority, permit 2021-01850.

Note that full information on the approval of the study protocol must also be provided in the manuscript.

Field-specific reporting

Please select the one below that is the best fit for your research. If you are not sure, read the appropriate sections before making your selection.

☒ Life sciences ☐ Behavioural & social sciences ☐ Ecological, evolutionary & environmental sciences

For a reference copy of the document with all sections, see nature.com/documents/nr-reporting-summary-flat.pdf

Life sciences study design

All studies must disclose on these points even when the disclosure is negative.

Sample size	Samples from two donors were utilized for lineage tracing. The number of donors was based off of availability of samples timed for detection of the peak plasmablast response.
Data exclusions	No data exclusion.
Replication	Three IgM repertoire sequencing libraries were generated per donor in order to verify reproducibility of the donor genotypes. Every replicate produced the exact same D and J genotypes, while V genotypes had an average jaccard index of 94.
Randomization	We performed an observational study of volunteers, therefore we did not plan a randomized trial.
Blinding	No condition or treatment was applied during this observational study, so blinding was not necessary.

Reporting for specific materials, systems and methods

We require information from authors about some types of materials, experimental systems and methods used in many studies. Here, indicate whether each material, system or method listed is relevant to your study. If you are not sure if a list item applies to your research, read the appropriate section before selecting a response.

Materials & experimental systems

n/a	Involved in the study
☐	☒ Antibodies
☐	☒ Eukaryotic cell lines
☒	☐ Palaeontology and archaeology
☒	☐ Animals and other organisms
☒	☐ Clinical data
☒	☐ Dual use research of concern

Methods

n/a	Involved in the study
☒	☐ ChIP-seq
☐	☒ Flow cytometry
☒	☐ MRI-based neuroimaging

Antibodies

Antibodies used

anti-human IgG-HRP (Thermo Fisher Scientific, Cat# 31413) detection antibody (1:8000)
PE-Cy7; Biolegend, Cat# 302216 (1:1000)
APC-Cy7; Biolegend, Cat#302313 (1:1000)
PE; Biolegend, Cat# 303506 (1:400)
PerCP-Cy5.5; Biolegend, Cat# 30040 (1:100)
PerCP-Cy5.5; Biolegend, Cat# 344710 (1:100)
PerCP-Cy5.5; Invitrogen, Cat# 45-0149-42 (1:100)
PerCP-Cy5.5; Biolegend, Cat# 360712 (1:100)
BV711; BD Biosciences, Cat# 747877 (1:100)
APC; Biolegend, Cat# 334107 (1:100)
BV510; BD Biosciences Cat# 740167 (1:100)
APC-Cy7; Biolegend, Cat# 334110 (1:100)

Validation

All antibodies were commercial, obtained from the Thermo Fisher Scientific, Biolegend, Invitrogen, and BD Biosciences companies, which verify the reactivity of their antibodies. These antibodies were not validated internally, as we relied on quality control checks performed by the manufacturer.

1. anti-human IgG-HRP (Thermo Fisher Scientific, Cat# 31413) detection antibody: Antibody specificity was demonstrated by detection of differential basal expression of IgG across cell lines owing to their inherent genetic constitution. Additionally, western blot was performed to confirm specificity to human IgG isotype and ELISA performed to screen purified IgG from donor serum against C. albicans yeast cell wall extract.

2. PE-Cy7; Biolegend, Cat# 302216

3. APC-Cy7; Biolegend, Cat#302313

4. PE; Biolegend, Cat# 303506 (1:400)

5. PerCP-Cy5.5; Biolegend, Cat# 300430 (1:100)

6. PerCP-Cy5.5; Biolegend, Cat# 344710 (1:100)

7. PerCP-Cy5.5; Invitrogen, Cat# 45-0149-42 (1:100): Manufacturer's note -"This 61D3 antibody has been pre-titrated and tested by flow cytometric analysis of normal human peripheral blood cells."

8. PerCP-Cy5.5; Biolegend, Cat# 360712 (1:100)

9. BV711; BD Biosciences, Cat# 747877 (1:100): Manufacturer's note - "The production process underwent stringent testing and validation to assure that it generates a high-quality conjugate with consistent performance and specific binding activity. However, verification testing has not been performed on all conjugate lots."

10. APC; Biolegend, Cat# 334107 (1:100)

11. BV510; BD Biosciences Cat# 740167 (1:100): Manufacturer's note - "The production process underwent stringent testing and validation to assure that it generates a high-quality conjugate with consistent performance and specific binding activity. However, verification testing has not been performed on all conjugate lots."

12. APC-Cy7; Biolegend, Cat# 334110 (1:100)

Validation statement on all Biolegend antibodies – "Each lot of this antibody is quality control tested by immunofluorescent staining with flow cytometric analysis."

Eukaryotic cell lines

Policy information about cell lines and Sex and Gender in Research

Cell line source(s)	Freestyle 293F - Purchased commercially from ThermoFisher, cat R79007<br>S. Cerevisiae - In-house strain<br>HEK-293 cells-STR analysis as performed by supplier (DSMZ, Germany), cat ACC 305<br>Hela-hACE2 cells - Purchased directly from BPS Bioscience, cat 79958<br>293T-hsACE2 cells - Purchased directly from Integral Molecular, cat C-HA102
Authentication	Freestyle 293F – Not authenticated internally<br>S. Cerevisiae - Not authenticated internally<br>HEK-293 cells-STR analysis as performed by supplier (DSMZ, Germany) Hela-hACE2 cells - Purchased directly from BPS Bioscience<br>293T-hsACE2 cells - Not authenticated internally
Mycoplasma contamination	All cell lines tested negative for mycoplasma contamination.<br>Freestyle 293F<br>S. Cerevisiae - In-house<br>HEK-293 cells<br>Hela-hACE2 cells - screened using the MycoAlertTM Mycoplasma Detection Kit (Lonza, #LT07-118) by supplier<br>293T-hsACE2 cells -tested with MycoStrip Mycoplasma Detection Kit, Sourced from InvivoGen (Cat#: rep-mys-10)
Commonly misidentified lines<br>(See ICLAC register)	No commonly misidentified lines were used in the study

Flow Cytometry

Plots

Confirm that:

☒ The axis labels state the marker and fluorochrome used (e.g. CD4-FITC).

☒ The axis scales are clearly visible. Include numbers along axes only for bottom left plot of group (a 'group' is an analysis of identical markers).

☒ All plots are contour plots with outliers or pseudocolor plots.

☒ A numerical value for number of cells or percentage (with statistics) is provided.

Methodology

Sample preparation	Frozen PBMCs were thawed and washed using FACS buffer (2%FBS, 1mM EDTA in PBS) followed by appropriate staining based on sample type. Acute samples post-infection and samples post-vaccination were stained to isolate antibody secreting cells (ASCs), whereas pre-vaccination PBMCs were stained for memory B cells (MBCs).<br>For ASC sorts, PBMCs were stained using anti-human CD19 (PE-Cy7; Biolegend, Cat# 302216), CD20 (APC-Cy7; Biolegend, Cat302313#),CD38 (PE; Biolegend, Cat# 303506), CD3 (PerCP-Cy5.5; Biolegend, Cat# 30040), CD8 (PerCP-Cy5.5; Biolegend, Cat# 344710), CD14 (PerCP-Cy5.5; Invitrogen, Cat# 45-0149-42), CD16 (PerCP-Cy5.5; Biolegend, Cat# 360712), IgM (BV711; BD Biosciences, Cat# 747877), CD71 (APC; Biolegend, Cat# 334107), CD27 (BV510; BD Biosciences Cat# 740167) and propidium iodide (PI). For MBC sorts, PBMCs were stained with CD19 (PE-Cy7; Biolegend, Cat# 302216), CD3 (PerCP-Cy5.5; Biolegend, Cat# 30040), CD8 (PerCP-Cy5.5; Biolegend, Cat# 344710), CD14 (PerCP-Cy5.5; Invitrogen, Cat# 45-0149-42), CD16 (PerCP-Cy5.5; Biolegend, Cat# 360712), IgM (BV711; BD Biosciences, Cat# 747877), CD71 (APC-Cy7; Biolegend, Cat# 334110), CD27 (BV510; BD Biosciences, Cat# 740167), PI and a freshly-prepared mixture of PE- and APC-labeled SARS-CoV-2 S-2P protein tetramers (25 nM each).<br>ASCs or antigen-specific MBCs were single-cell index sorted using a BD FACS Aria II Fusion (BD Biosciences) into 96-well

	polypropylene microplates (Corning Cat# 07-200-95) containing 20 µl/well of lysis buffer [5 µl of 5X first strand SSIV cDNA buffer (Invitrogen Cat # 18090050B), 0.25 µl RNaseOUT (Invitrogen Cat#10777019), 0.625 µl of NP-40 (Thermo Scientific Cat# 85124),1.25 µl dithiothreitol (Invitrogen), and 12.85 µl dH2O]. Plates were spun down at 1,000 × g for 30 s and stored at -80° C until use.
Instrument	BD FACS Aria II Fusion (BD Biosciences).
Software	FlowJo Version 10.8.1
Cell population abundance	Purity was determined by specific and relevant staining of markers using flow cytometry. Additionally, single-cell index sorting data confirmed the purity of sorted population. For ASC sorts, 1.8-2 million and for MBCs, 0.6-3 million total events were collected.
Gating strategy	All samples were FSC-A/SSC-A gated followed by FSC-A/FSC-H and SSC-A/SSC-H gating for singlet cells. Subsequent relevant gating involved dead cell exclusion and B cell inclusion, defined by PI- and CD19+, respectively. ASCs were defined as CD19+CD20loCD38+CD27+CD3−CD8−CD14−CD16−PI− and antigen-specific MBCs were defined as CD19+CD3−CD8−CD14−CD16−PI−IgM−IgD− cells that showed reactivity to both SARS-CoV-2 S-2P tetramers. Naïve, pre-pandemic samples were used to set the thresholds for antigen reactivity.

☒ Tick this box to confirm that a figure exemplifying the gating strategy is provided in the Supplementary Information.
